# Can negative life events and coping style help explain socioeconomic differences in perceived stress among adolescents? A cross-sectional study based on the West Jutland cohort study

**DOI:** 10.1186/1471-2458-13-532

**Published:** 2013-06-02

**Authors:** David J Glasscock, Johan H Andersen, Merete Labriola, Kurt Rasmussen, Claus D Hansen

**Affiliations:** 1 Danish Ramazzini Centre, Department of Occupational Medicine, Regional Hospital Herning, Herning, Denmark; 2 Department of Clinical Social Medicine, Public Health and Quality Management, Central Denmark Region and Section of Clinical Social Medicine & Rehabilitation, School of Public Health, University of Aarhus, Aarhus, Denmark; 3 Department of Sociology and Social Work, Aalborg University, Aalborg, Denmark

**Keywords:** Social gradient, Perceived stress, Life events, Coping, Adolescence

## Abstract

**Background:**

Previous research suggests that perceived stress in adolescence is socially patterned, but that this relationship may depend on the measure of socioeconomic status (SES) used. This study examines if social gradients in perceived stress, negative life events, and coping exist amongst Danish adolescents, and, if life events and coping strategies can partly account for an association between SES and perceived stress. These relationships are studied separately for two different measures of SES.

**Methods:**

Questionnaire data were collected from 3054 14–15 year old youths (83% response rate) during baseline measurement in the West Jutland birth cohort study. Parents were identified via the Central Office of Civil Registration in which the respondents are linked to their parents or guardians via their CPR-number, a personal identification number given to everyone in Denmark. The study employs data from two independent sources, adolescent self-report data (stress, life events and coping) and national registers (parental educational level, household income and confounder variables). Ordinary Least Squares regression estimated the effects of parental SES, negative life events and coping on perceived stress. Analyses were stratified by gender.

**Results:**

Girls reported more perceived stress than boys. SES accounted for a small but significant amount of the variance in perceived stress. Lower parental education and lower household income were associated with higher stress levels irrespective of gender, but the social gradient was strongest amongst girls when parents’ education was used to measure SES, and strongest for boys when income was used. Life events and coping were also found to be associated with SES and both mediated part of the SES-perceived stress relationship. In general, the social gradient in perceived stress was accounted for by the study variables to a higher degree among girls than among boys.

**Conclusions:**

Lower parental education and household income are associated with higher levels of perceived stress amongst Danish adolescents. Furthermore, both life events and coping appear to mediate this relation. Gender differences in the ways SES and stress are related may exist.

## Background

Associations between health and socioeconomic status (SES) are well documented in many countries [1-3], including Denmark [4-7]. These associations often take the form of a social gradient in health whereby each positive increment in social position, ie. better education or higher income, is related to a reduced risk of illness. The higher the status in the social hierarchy, the healthier a person is likely to be. This social gradient in health has also been referred to as the status syndrome [8]. Many models and theories have been suggested as explanations for such social inequalities in health, amongst them psychosocial pathways and stress mechanisms [9-14]. One of these, the reserve capacity model, proposed by Gallo & Mathews [13], acts as the theoretical framework or heuristic for the ‘West Jutland Cohort Study’. ‘The West Jutland Cohort Study’ is a longitudinal follow-up study of the social gradient in health which focuses particularly on psychosocial pathways that may connect SES and physical health outcomes throughout the life course. This paper presents analyses of data from the baseline year of this ongoing study, when the cohort is in its adolescent years.

The general framework proposed by Gallo & Mathews [13] aims at understanding the roles of cognitive-emotional factors during the life course that potentially link SES with physical health. The model suggests that low-SES environments are stressful and reduce individuals’ reserve capacity to manage stress, thereby increasing vulnerability to negative emotions and cognitions. In other words, the lower a person’s SES, the greater the exposure to stressful events and conditions, and the longer the exposure, the more the individual’s reserve capacity and ability to cope with stress will be eroded. The model suggests cumulative effects over long time-spans. In the course of time chronic stress influences physical health indirectly via health behaviours and directly via changes in metabolic functioning. In our adolescent sample the focus is not on physical health outcomes but rather on some of the psychosocial factors proposed by Gallo & Mathews [13] to mediate the SES – health relationship. More specifically, we examine if a social gradient in stress can be detected at this early age and if so, whether differential exposure (negative life events) and/or differential vulnerability (coping) can help explain this.

### Existing research on negative life events, coping style and perceived stress among adolescents

It is well established in adult populations that the socially disadvantaged are more likely to experience symptoms of mental distress [15]. Findings of greater exposure to stressors such as life events in lower as compared to higher SES-groups have also been reported [15-18]. Orpana and Lemyre [19] found that exposure to stressors, including negative life events, accounted for a large proportion of the relationship between income group and poor self-rated health among adults in Canada, especially for men. Lantz et al. [20] also present data, this time from a prospective study, supporting the hypothesis that differential exposure to stress and negative life events is one way in which socioeconomic inequalities in health are produced.

Coping resources that may influence how individuals react to stressful events and conditions once they arise have also been found to be unequally distributed between adult SES-groups. For example, a positive relationship with SES has been reported for belief in personal control or mastery [21-23], optimism [24], social support [25]. However, evidence for a relation between actual coping strategies and SES is meagre [24]. The coping literature often distinguishes between active and avoidant coping strategies (ibid). Using active coping strategies is generally thought to be a better and more adaptive way of dealing with stressful events, while avoidance coping is considered less adaptive [26].

Research on social differences in psychological stress and factors related to its cause has usually focussed on adult populations. Only recently has research on social disparities in stress amongst adolescent populations appeared. Findings of a social gradient that indicate poorer health amongst lower SES-groups have been consistent for many health outcomes in both child and adult populations. Amongst adolescents the picture is less clear cut. Some studies with teenage populations, including Danish ones, find social gradients in both physical and psychological symptoms [27-30], while others find no or very weak gradients in this age group [31-33].

Goodman et al. [34] suggest that adolescence is a critical developmental period for understanding how social characteristics such as SES, which are linked to social disadvantage, are related to the development of poor health. They present analyses showing higher levels of perceived stress among adolescents from lower SES families. However, this was the case when parental education was used as a measure of SES, but not household income. Finkelstein et al. [35] examined possible mechanisms of the relationship between SES and stress, focusing on psychological resources. They found that optimism partially mediated the relation between SES and stress. Adolescents from lower SES backgrounds were more pessimistic, while pessimism increased the risk of high stress.

Wills et al. [36] report that lower SES (parental education) was associated with greater substance use and more negative life events. Furthermore, the relation between SES and substance use was partly mediated by stressful life events, but not coping. Gore et al. [37] also report a higher frequency of life events for lower SES ninth through eleventh grade adolescents.

Among Danish adolescents, Nielsen et al. [38] did not find a clear relationship between SES (Family Affluence scale [39]) and stress. For boys a medium level of family affluence was associated with the lowest stress levels, while low family affluence was associated with the highest stress levels. No relationship was found for girls. However, a greater frequency of stressful life events was found for adolescents with lower Family Affluence.

An important issue in research on relations between SES and health or stress is the conceptualisation and measurement of SES. SES has been measured in many different ways and there remains a lack of consensus concerning the extent to which these measures (e.g. occupational status, income, education, composite measures combining two or more of these) tap into the same construct, different aspects of the same construct, or represent separate entities. One possible explanation for mixed research results could be the use of different SES measures that are assumed to be alternatives. But research suggests that different measures of SES, such as income and education, affect health through different pathways, for which reason combining them is not recommended [40-43]. For example, Goodman [28] reports findings showing that income is related to adolescent suicide attempts, while parental education and occupation is not. Other researchers have found that the extent to which income and parental education are correlated with each other depends on other factors such as ethnic background [44]. In a Danish setting, we found that parental education was associated with obesity while household income was not. In that paper, we employed a Bourdieu-inspired argument for using household income (economic capital in Bourdieu’s terminology) and educational level in the household (a proxy for ‘cultural capital’) as the two most important measures of SES [7]. The main argument Bourdieu provides is his idea that modern societies are primarily stratified by two independent yet interrelated mechanisms: economic capital and cultural capital [45]. These are likely to be linked to health and in this case perceived stress in different ways: having low levels of economic capital could make a person more prone to experience negative life events and living in situations that are more stressful e.g. instance due to lack of material resources. On the other hand, lower levels of cultural capital e.g. education and knowledge would make a person less susceptible to make use of health related information and influence the way in which they cope with stressful situations. There are clearly good reasons for using more than one measure of SES within the same study so that eventual differences in how they are associated with stress or health related outcomes can be uncovered and examined. This approach is employed in the present study. Given the present state of research, it seems likely that different aspects of SES are related to adolescents’ experience of stress and that mechanisms are complex and varied. The use of both income and education in this paper thus tries to take into account both aspects of the socioeconomic differentiation of modern societies.

### Aims of this paper

The purpose of the cross-sectional analyses presented here is to contribute to the sparse literature on social disparities in stress among adolescents. We examine if negative life events and coping mediate the relationship between SES and perceived stress. To do this we address the following research questions: a) Is a social gradient in perceived stress already present in this age-group? b) Is there a social gradient in the number of negative life events experienced during childhood? c) Is SES related to differences in the ways adolescents cope with stress? d) Are life events and coping associated with perceived stress? e) Do life events and/or coping mediate the relation between SES and perceived stress? f) Are these relations affected by the choice of SES-measure employed?

## Methods

### Data

The data is taken from the first round of the West Jutland cohort, a birth cohort consisting of all individuals born in 1989 living in the county of Ringkjøbing, Denmark, primo April 2004. Baseline data was collected in 2004 when the birth cohort was 14–15 years old. The study setting is a predominantly rural area of Denmark, where the occupational structure is dominated by industry, commerce and agriculture. As can be seen from Table 1, the social structure is comparable to the rest of Denmark, although families in this part of the country are less likely to have a long educational background than in more urbanized areas. Average disposable household income for members of the cohort is virtually identical when they are compared to peers of the same age from other parts of Denmark using register based information. More adolescents from the West Jutland Cohort Study live with parents who are married. Compared with non-Scandinavian countries differences in individual income are considerably reduced due to a progressive taxation system. Educational differences are comparable to most Western countries.

**Table 1:** Sociodemographic characteristics of Ringkjøbing County compared to the rest of Denmark 2003

	**West Jutland Cohort Study**	**Rest of Denmark**
	**(n = 3677)**	**(n = 60889)**
**Gender (in %)**		
Boys	51.7	51.3
Girls	48.3	48.7
**Family income**		
Mean disposable income (Dkr)	163717	168550
**Highest household education (in %)**		
Primary school	20.3	15.2
Vocational training	4.8	2.4
University degree	2.9	6.3
**Cohabitation status (in %)**		
Living alone	1.9	2.2
Living with married parents	75.2	66.2
Living with cohabitating parents	10.4	11.5
Living with lone parents	12.5	20.1

All schools in the study area were contacted and asked to permit the research team to administer questionnaires to the pupils in class. This approach was employed to ensure a high response rate. Permission was obtained from most schools and 65% of the completed questionnaires were obtained in this way during May 2004. For those not present (or for those attending schools who did not permit access to the research team) questionnaires were sent by mail.

The cohort comprises 3,681 individuals of which 3,054 answered the questionnaire resulting in a response rate of 83%. Parents were identified using Central Office of Civil Registration in which the respondents are linked to their parents or guardians via CPR, a personal identification number given to everyone in Denmark at birth (or upon entry for immigrants). CPR was also used to link information from the questionnaires to official registers held at Statistics Denmark. The study and the linking of information across registers were approved by the Danish Data Protection Agency (Study No. 2009-41-3761).

The study thus employs data from two independent sources, adolescent self-report data (stress, life events and coping) and national registers (SES and confounder variables).

An analysis of the non-responders showed that they on average had poorer and less educated parents and that they were more likely to have a non-Danish ethnic background.

### Measures

All the measures were translated into Danish by the research group using the translation/back-translation procedure recommended by Brislin [46]. The translation/back-translation process was carried out by three individuals independently of each other and in the event of disagreement the items were discussed and a final decision was agreed upon. The questionnaire was tested in a pilot study by administering it to groups of 14–15 year olds and subsequently conducting interviews to ensure correct understanding and receive feedback on suggested changes.

#### Perceived stress

Perceived stress was measured using the 4 item version of Cohen’s Perceived Stress Scale (PSS) which has been found to be a good measure of the extent to which individuals perceive their situation to be unpredictable and without control [47]. Higher scores indicate more stress. In this sample the scale had Cronbach’s alpha of 0.62. This is identical to the coefficient (0.60) reported by Cohen & Williamson [48]. The scale is used as a continuous variable in the analyses after performing check for normality.

#### Negative life events

Negative life events were measured by 6 items taken from Newcomb, Huba, & Bentler’s [49] measure and the Social Stress Indicator [50]. The wording of the question was the following: “In your life-time”: 1: “Have your parents divorced?” 2: “Have you lost any of your parents because they died?” 3: “Have any of your parents abused alcohol or drugs to an extent where it caused problems in the family?” 4: “Have you been abused by someone you knew?” 5: “Have you witnessed a very violent event?” and 6: “Have your parents suffered a life-threatening disease or accident?”. Response options were yes/no and were summated for each individual yielding an index score between 0 and 5 since none of the participants indicated to have experienced all six negative life events. The variable was used as a continuous variable in the analyses in order to be able to include in the mediational analyses. Analysing the variable as a categorical variable showed a monotonically increasing trend in the beta coefficients for higher number of negative life events which supports the use of the variable as a continuous predictor. We considered using register-based information to create an indicator of parental death and divorce. However, we decided to use the self-report data instead because combining the measures would be difficult. Most importantly, however, the register based data may not reflect the actual relationship the adolescents have to their parents or those they consider their parents (e.g. a step-mother or –father they are more emotionally connected to than their biological or legal parents which may be the only thing that can be derived from the registers).

#### Coping

Coping was measured using 5 subscales of two items each from the Brief COPE Scale [50]. Each item had four response categories yielding scores on each item between 1 and 4. The decision not to include all 14 subscales from this measure was based on considerations about questionnaire length. The subscales used here were chosen partly for their appropriateness in a Danish youth context, partly on the basis of Cronbach alpha coefficients reported by Carver [51]. The subscales employed in this paper are ‘active coping’, ‘planning’, ‘positive reframing’, ‘self distraction’, and ‘behavioural disengagement’. Cronbach’s Alpha for the subscales ranged from 0.49 to 0.86. This was similar to the α values reported by Carver supporting the adequacy of our translation. To simplify analyses we rationally grouped the 5 subscales into two coping dimensions that emphasise either an ‘active’ approach to problem solving, generally considered to be more adaptive, or an ‘avoidance’ based approach, considered to be less adaptive. Other researchers have adopted a similar approach when using the Brief COPE Scale, e.g. Schnider et al. [52]. The 6 items from the subscales ‘Active coping’, ‘planing’ and ‘positive reframing’ were combined to form the ‘active’ coping scale (Cronbach’s Alpha = 0.75) and the 4 items from the subscales ‘self-distraction’ and ‘behavioural disengagement’ were used to form the ‘avoidance’ coping scale (Cronbach’s Alpha = 0.53). Both scales were created taking the mean of the items thus yielding two scales with scores between 1 and 4 where higher scores indicates higher levels of that type of coping.

#### Socioeconomic status

To measure SES we used information about the participants’ parents from national registers on income and education in 2003 (i.e. the year before data was collected). We used household income as one measure of SES and highest attained educational level in the household as another. In those cases where the participants’ parents had been divorced we used information on the household in which the participants had their place of residence according to CPR. Information on income was taken from the tax register (recoded into tertiles for some of the analyses) and information on educational attainment was taken from the Danish Educational Register and recoded into four categories: compulsory school (< 10 years of education), high school/vocational training (10–12 years), short and medium term higher education (called KVU and MVU in Danish) (12–15 years), university degree or equivalent (15 years or more).

#### Confounder variables

In order to take into account factors that could confound potential associations between SES and perceived stress we included three confounders: cohabitation status of parents in the household (lone parent vs. cohabitating), number of siblings (coded into 4 categories), and ethnic background (Danish parents vs. at least one immigrant parent). These variables might affect household income and parental education. These data were taken from national registers.

### Statistical analyses

In order to describe the study sample, mean scores on the four key variables (perceived stress, number of negative life events, avoidance and active coping) are reported for each of the independent variables (i.e. the two measures of SES). The results of these analyses are presented in Table 2. Ordinary Least Squares (OLS) regression was used to estimate the associations between perceived stress, parental SES, negative life events and coping. Residuals were checked to see whether the data were in concordance with the assumptions behind OLS (i.e. multivariate normality, homoscedasticity). The Q-norm plot showed a slight deviation from the normality assumption but excluding cases with the highest residuals (Studentized residuals >3) did not change the associations substantially (i.e. all changes in the beta coefficients were below 0.03). Robust standard errors were computed, but they did not change the results. In order to assess whether the association between parental SES and adolescent perceived stress is mediated by negative life events and coping, the analysis was carried out in four steps entering different variables at each step. The variables entered at each step can be seen from the notes in the tables. The proportion of the original association explained by coping and life events respectively was calculated as ((β_Crude_ - β_Adjusted_)/(β_Crude_) * 100). For education this was calculated entering the variable as a linear term and comparing the β values before and after adjusting for the two SES measures. In order to test the statistical significance of the mediation Sobel’s tests were carried out on each of the mediators [53] using the sgmediation command in STATA. The analyses were stratified by gender as a consequence of the gender differences found in Table 2. After exclusion of all those with missing data on any of the variables used in the multivariate analysis (n = 157), the sample used for the analyses consisted of 2901 respondents, 1445 boys and 1456 girls. Analyses were performed using STATA v12.

**Table 2:** Mean levels (including 95% Confidence Intervals) of perceived stress, number of life events, active and avoidance coping for adolescents by parents’ highest education and household income

		**Perceived Stress**		**Negative life events**		**Active coping**		**Avoidance coping**	
		**(range: 0–16)**		**(range: 0–5)**		**(range: 1–4)**		**(range: 1–4)**	
		**Boys**	**Girls**	**Boys**	**Girls**	**Boys**	**Girls**	**Boys**	**Girls**
		4.87 (4.75-4.99)	5.64 (5.51-5.78)	0.54 (0.50-0.59)	0.65 (0.60-0.69)	2.65 (2.62-2.68)	2.62 (2.60-2.65)	1.94 (1.92-1.97)	1.97 (1.94-1.99)
**Highest educational level in household (2003)**	**N (percent)**	p = 0.071	p = 0.003	p = 0.002	p < 0.000	p = 0.167	p = 0.035	p = 0.669	p = 0.004
Primary school (< 10 years)	229 (10.2)	5.35 (4.97-5.74)	6.46 (6.08-6.84)	0.83 (0.68-1.00)	1.06 (0.89-1.22)	2.66 (2.56-2.75)	2.60 (2.53-2.68)	2.04 (1.96-2.13)	2.04 (1.96-2.11)
High School/Vocational training (10–12 years)	1169 (52.0)	4.81 (4.64-4.98)	5.59 (5.41-5.77)	0.53 (0.47-0.59)	0.59 (0.53-0.66)	2.62 (2.58-2.66)	2.59 (2.56-2.63)	1.93 (1.89-1.96)	1.99 (1.96-2.02)
Higher education (KVU/MVU) (13–15 years)	704 (31.3)	4.76 (4.54-4.97)	5.46 (5.21-5.70)	0.41 (0.35-0.48)	0.50 (0.43-0.57)	2.67 (2.62-2.72)	2.65 (2.60-2.69)	1.92 (1.88-1.97)	1.91 (1.87-1.95)
University or equivalent (> 15 years)	148 (6.6)	4.49 (4.03-4.94)	5.07 (4.57-5.57)	0.31 (0.19-0.43)	0.44 (0.29-0.59)	2.74 (2.63-2.85)	2.79 (2.68-2.90)	1.98 (1.87-2.10)	1.93 (1.84-2.01)
**Household income (2003)**		p < 0.000	p = 0.004	p < 0.000	p < 0.000	p = 0.234	p = 0.003	p = 0.072	p = 0.472
1. tertile (< 77,382 US $)	593 (26.1)	5.42 (5.18-5.67)	5.86 (5.63-6.09)	1.03 (0.93-1.13)	1.13 (1.02-1.23)	2.59 (2.53-2.64)	2.65 (2.60-2.69)	2.02 (1.97-2.06)	2.00 (1.96-2.05)
2. tertile (77,382-100,531 US $)	805 (35.4)	4.77 (4.57-4.98)	5.86 (5.63-6.10)	0.41 (0.35-0.47)	0.53 (0.46-0.60)	2.66 (2.61-2.70)	2.55 (2.51-2.60)	1.91 (1.87-1.96)	1.97 (1.93-2.01)
3. tertile (>100,531 US $)	879 (38.6)	4.55 (4.36-4.74)	5.26 (5.05-5.48)	0.31 (0.26-0.36)	0.35 (0.29-0.40)	2.68 (2.64-2.73)	2.66 (2.62-2.71)	1.92 (1.88-1.96)	1.94 (1.90-1.97)

P-values indicate test for mean differences across the two SES- measures estimated separately for boys and girls.

Stratified by gender.

## Results

Table 2 contains descriptive statistics of the variables used in the analyses. Girls report significantly higher levels of perceived stress than boys do (p < 0.001). For that reason we decided to stratify the analyses by gender in case there were substantial differences in the relation between SES and perceived stress as well as the way in which the two measures of SES were mediated by negative life events and coping.

There is a consistent social gradient in perceived stress amongst both boys and girls regardless of the measure of SES one uses, although the amount of variance in perceived stress accounted for by SES is small (r^2^ < 0.03 for both measures of SES). Lower parental education and lower household income are associated with higher stress levels. However, it would seem that the gradient is strongest amongst girls if parents’ education is used as the measure of SES, while income has the strongest gradient for boys. There is a clear social gradient for negative life events. Each downward step in SES is connected with an increased number of life events during childhood. This is not specific to the SES measure used or to gender, though girls report more events than boys (p = 0.002). This may in part be caused by slightly more girls experiencing their parents being divorced. While there is no gender difference in the amount of coping used and the SES differences in coping style is rather weak as well. For girls, higher parental education is associated with more active coping and less avoidance coping. These relationships do not reach significance for boys. On the other hand, boys show a non-significant trend regarding active coping. Increased income is connected with more active coping. This relationship is absent for girls.

Tables 3 and 4 show the associations between the two SES measures and perceived stress, when entering the different mediator and confounder variables in separate steps in the regression analyses, for boys and girls respectively.

**Table 3:** Perceived stress and its association with SES adjusted for negative life events and coping among boys

	**Model 1**			**Model 2**			**Model 3**			**Model 4**		
	β	Std. β	SE	β	Std. β	SE	β	Std. β	SE	β	Std. β	SE
**Household income (in 1000 Dkr)**	-0.001***	-0.106	0.000	-0.001**	-0.089	0.000	-0.001**	-0.086	0.000	-0.001**	-0.078	0.000
*% of total income effect mediated*	*-*			*15.6%*			*18.9%*			*36.9%*		
				*(Sobels test = 3.22, p < 0.01)*			*(Sobels tests = 0.37, p = 0.71)*			*(Sobels test = 5.66, p < 0.01)*		
**Parental education**												
Primary school (< 10 years)	reference			reference			Reference			reference		
High School/vocational training (10–12 years)	-0.399	-0.084	0.211	-0.360	-0.125	0.212	-0.304	-0.064	0.195	-0.267	-0.056	0.192
Higher education (kvu/mvu) (13–15 years)	-0.425	-0.084	0.222	-0.365	-0.130	0.229	-0.287	-0.057	0.205	-0.231	-0.046	0.202
University (> 15 years)	-0.739*	-0.071	0.326	-0.578	-0.103	0.333	-0.605*	-0.058	0.301	-0.451	-0.043	0.298
*% of total educational effect mediated*	*-*			*22.8%*			*28.2%*			*50.0%*		
				*(Sobels test = 2.70, p < 0.01)*			*(Sobels test = 2.90, p < 0.01)*			*(Sobels test = 4.15, p < 0.01)*		

* .05 > p > .01; ** .01 > p > .001; *** p ≤ .001.

Ordinary Least Squares regression. (n = 1445).

For each SES measure (i.e. household income and parental education) the following four models where carried out adjusted for confounders (no. siblings, parents cohabitation status & ethnicity):

Model 1: Individual SES measure and perceived stress.

Model 2: Individual SES measure + life events.

Model 3: Individual SES measure + active and avoidance coping.

Model 4: Individual SES measure + life events, active and avoidance coping.

**Table 4:** Perceived stress and its association with SES adjusted for negative life events and coping among Girls

	**Model 1**			**Model 2**			**Model 3**			**Model 4**		
	β	Std. β	SE	β	Std. β	SE	β	Std. β	SE	β	Std. β	SE
**Household income (in 1000 Dkr)**	-0.001**	-0.095	0.000	-0.001**	-0.081	0.000	-0.001*	-0.059	0.000	0.000	-0.050	0.000
*% of total income effect mediated*	*-*			*14.7%*			*37.7%*			*49.2%*		
				*(Sobels test = 2.99, p < 0.01)*			*(Sobels test = 15.46, p < 0.01)*			*(Sobels test = 11.97, p < 0.01)*		
**Parental education**												
Primary school (< 10 years)	reference			reference			reference			reference		
High School/vocational training (10–12 years)	-0.734**	-0.142	0.213	-0.646**	-0.125	0.212	-0.688***	-0.134	0.189	-0.618**	-0.120	0.189
Higher education (kvu/mvu) (13–15 years)	-0.856***	-0.150	0.230	-0.743**	-0.130	0.229	-0.612**	-0.107	0.205	-0.524*	-0.092	0.204
University (> 15 years)	-1.265***	-0.117	0.334	-1.115**	-0.103	0.333	-0.815**	-0.075	0.298	-0.697*	-0.064	0.297
*% of total educational effect mediated*	*-*			*13.3%*			*45.9%*			*56.3%*		
				*(Sobels test = 3.08, p < 0.01)*			*(Sobels test = 24.18, p < 0.01)*			*(Sobels test = 17.98, p < 0.01)*		

* .05 > p > .01; ** .01 > p > .001; *** p ≤ .001.

Ordinary Least Squares regression. (n = 1456).

For each SES measure (i.e. household income and parental education) the following four models where carried out adjusted for confounders (no. siblings, parents cohabitation status & ethnicity):

Model 1: Individual SES measure and perceived stress.

Model 2: Individual SES measure + life events.

Model 3: Individual SES measure + active and avoidance coping.

Model 4: Individual SES measure + life events, active and avoidance coping.

The beta coefficients in model 1 show the impact of the two SES measures on perceived stress for each gender. The results show that SES differences are rather small but income seems to be more important for boys while education is correlated stronger with perceived stress in girls. The decrease in the β values for household income and parental education in model 2 indicates that the SES – perceived stress relation is partly mediated by life events. This is confirmed by the Sobel tests for model 2 in both Tables 3 and 4. After inclusion in the model of life events, education is no longer significantly associated with stress among boys (Table 3). Comparing the crude β with the adjusted indicates that life events mediate between 13% and 23% of the original SES differences in perceived stress. In model 3, life events are replaced by the two coping variables. This produces larger beta coefficients than was seen in model 2, indicating that coping is more strongly associated with perceived stress than life events. The proportion of the SES differences in perceived stress mediated by coping is of the same magnitude (approximately 18%) as was the case for life events amongst boys and substantially higher for girls (46% vs 13%). For girls, but not boys, a larger proportion of the SES-stress gradient is accounted for by coping than by life events when parental education is the indicator of SES. Overall, the total SES differences mediated by negative life events and coping are somewhat higher among girls (49% and 56%) than is the case for boys (37% and 50%).

In Table 5 associations between the mediator variables and perceived stress are examined, both with and without controlling for SES and confounders. We can see that the strongest associations are between coping and stress. Coping appears to be more strongly related to perceived stress among girls. For both sexes, higher levels of active coping are related to less stress, while higher levels of avoidance coping are related to more stress. Negative life events are also clearly related to stress. More life events are predictive of higher stress levels. There is no gender difference in the strength of the association. The main gender difference here seems to be the association between active coping and stress, which is strongest for girls. This is also confirmed when running a regression analysis on the whole sample incorporating an interaction effect between gender and active coping (p < 0.000). Apart from the association between life events and perceived stress amongst boys, the confounder variables do not seem to attenuate the observed associations (see Table 5, model 3).

**Table 5:** Perceived stress and its associations with coping and negative life events

	**Model 1**			**Model 2**			**Model 3**		
	β	Std. β	SE	β	Std. β	SE	β	Std. β	SE
**Boys**									
**Life events**	0.587^***^	0.196	0.078	0.503^***^	0.168	0.072	0.535^***^	0.091	0.091
**Active coping**	-1.129^***^	-0.258	0.111	-0.958^***^	-0.219	0.105	-0.948^***^	-0.105	0.105
**Avoidance coping**	1.596^***^	0.328	0.121	1.410^***^	0.290	0.117	1.407^***^	0.117	0.117
**Girls**									
**Life events**	0.531^***^	0.186	0.073	0.435^***^	0.153	0.066	0.398^***^	0.140	0.087
**Active coping**	-1.895^***^	-0.375	0.123	-1.638^***^	-0.324	0.118	-1.637^***^	-0.324	0.118
**Avoidance coping**	1.874^***^	0.332	0.140	1.479^***^	0.262	0.132	1.448^***^	0.256	0.132

* .05 > p > .01; ** .01 > p > .001; *** p ≤ .001.

Ordinary Least Squares regression. Stratified by gender.

For all mediating variables (i.e. Life events, Active coping and Avoidance coping) the following models were tested:

Model 1: Bivariate associations between each mediating variable and perceived stress.

Model 2: Associations between each mediating variable and perceived stress adjusted for other mediating variables.

Model 3: Fully adjusted model i.e. all mediating variables, both SES measures, no. siblings, parents cohabitation status & ethnicity.

## Discussion

### Overall findings

In terms of the research questions posed in the introduction, the main results of the study can be summarized as follows: a) There is a consistent social gradient in perceived stress irrespective of gender or the SES measure used. In general, adolescents with lower SES report higher levels of perceived stress. However, the amount of variance in perceived stress accounted for by SES is very small. b) There is also a social gradient in the number of negative life events experienced during adolescence, and c) the ways Danish adolescents attempt to cope with stress appears to be socially patterned. d) Life events and, to a greater extent, coping are associated with perceived stress. e) Furthermore, both life events and coping partly mediates the SES differences in perceived stress. When both life events and coping are included in the model between 37 and 56% of the social gradient in perceived stress is mediated, depending on gender and the measure of SES employed. Sobel’s tests confirmed the existence of mediation for all the associations with the exception of coping style as a mediator of income differences in perceived stress for boys. f) Finally, the analyses presented here indicate that different measures of SES are related to perceived stress in slightly different ways, that is, via different mediational pathways. In general, parental education appears to have more influence on female adolescents’ stress, while household income appears to have more influence on male adolescents’ stress.

### SES and perceived stress

It was mentioned in the introduction that research into SES differences in stress amongst adolescents has been sparse, but that others have found higher levels of perceived stress among adolescents from lower SES families. Goodman et al. [34] report a similar finding, although in their sample, the association was only present when parental education was the indicator of SES, and absent when household income was employed. Both measures of SES were related to perceived stress in our sample, though the amount of variance in perceived stress accounted for by SES was very small. It is likely that both the amount of variance accounted for and the question of which SES-markers will be related to stress will be affected by contextual factors in the area in which a study is undertaken. It is possible, for example, that the amount of variance that can be accounted for will be larger in countries with a less egalitarian welfare system than that found in Denmark. Another possible explanation of the rather small differences in perceived stress across SES could be the inadequacy of tapping into actual status hierarchies influencing adolescents’ psychological well-being using information on parental status. It may be that the equalization of SES-differences in adolescents across a range of different measures of health [33] is caused by inadequate measures of SES among adolescents because their socioeconomic status is partly caused by the resources provided to them by their parents but is partly influenced by their own prestige and status in peer groups where different mechanism might be working (e.g. popularity due to sports achievements, intelligence, physical attractiveness etc.). If this is the case only the use of more subjective measures of SES could remedy this. Others have also reported higher frequencies of negative life events amongst lower SES adolescence [e.g. [36,37,54]].

### SES and coping

Research into associations between SES and coping strategies is meagre [24]. Wills et al. [36] examined associations between SES and many different coping variables, amongst them ‘helpless coping’, which consisted of items from Carver et al’s [55] disengagement coping scale. Items from this scale constitute 50% of the items in our avoidance coping scale. ‘Helpless coping’ was found to be significantly related to parental education; higher parental education was related to lower levels of helplessness, which is in line with our results. More adaptive forms of coping were found to be positively related to parental education.

Both life events and coping were, as expected, related to perceived stress levels. There are at least two possible explanations of why coping was more strongly associated with perceived stress than were life events. The first explanation concerns temporal proximity. While the life events measured here occurred throughout childhood, our measure of coping concerns how adolescents generally attempt to manage stress in the present. Coping is therefore closer in time to present stress levels than are life events. Many mediating and moderating variables intercede between initial stressors, such as life events, occurring during the course of life, and present day stress levels. Coping styles are likely to be the result of expectations of control, which in turn are shaped by previous experiences of success and failure in dealing with the challenges of life and an awareness of available resources. Coping is “farther along on the psychosocial chain as a mediator” [24]. Secondly, there is likely to be a greater degree of causal reciprocity between coping and perceived stress than between life events and perceived stress. That is, stress levels are not purely a result of coping, but also something that partly determine the choice of coping strategy. When stress levels are higher, as they are for lower SES adolescents, this will often reflect expectations of inadequate control, which is likely to promote avoidance rather than attempts to change the (uncontrollable) situation.

### Life events and coping as mediators

The main aim of the present study was to examine the extent to which life events and coping mediated SES-differences in perceived stress. In the present study, both life events and coping mediated some of these differences. For both boys and girls coping appear to be a stronger mediator than life events, irrespective of which SES-indicator is employed. In general, coping mediates SES-differences in perceived stress to a larger degree among girls than boys. This is most apparent for the gender difference in the effect of household income mediated by coping where 37% of the income differences in SES are mediated among girls and the mediation effect is not statistical significant for boys. These differences cannot be explained by the data available. It is possible that gender differences in socialisation and maturational processes play a part. That is, girls of this age may be psychologically more mature on average than their male peers, which might affect the extent to which their coping reflects strategies and habits that are learned from their parents. This may therefore be a very age-specific gender difference. It is also possible that girls for some reason are more sensitive to childhood conditions connected with parental education, that parental education has a more formative influence on coping for girls, while boys’ coping strategies are more influenced by material conditions reflecting household income. Perhaps higher parental education seems to promote more active coping and less avoidance coping.

Unfortunately there is very little in the literature that can help unravel such speculations. We have found only one previous study that investigates possible mechanisms connecting SES with perceived stress among adolescents by looking for mediation effects. Using cross-sectional analyses, Finkelstein et al. [35] examined the extent to which optimism and adaptive coping influence the relation between parental education and perceived stress. SES was related to stress, optimism and ‘disengagement coping’, but not ‘engagement coping’. Higher optimism and engagement coping were also related to less stress, while higher disengagement coping was related to more stress. Conceptually these coping measures appear very similar to the distinction in the present study between active coping and avoidance coping. Engagement coping and active coping entail attempts to deal directly with problems that one acknowledges, and is considered more adaptive, while disengagement and avoidance coping involve cognitions and behaviours that aim at creating distance to problems which one tries not to acknowledge. However, unlike in the present study, adding the coping variables to the regression model did not attenuate the effect of SES on stress. While optimism was found to mediate the SES – perceived stress relationship, no evidence of a meditational role for coping was found. This may reflect the fact that relations between coping and stress and between SES and coping appear weaker in Finkelstein et al’s study than in ours. The age range in Finkelstein et al’s sample was larger (12–20 years) compared with our birth cohort. Since coping behaviours are likely to change quite dramatically from year to year during this crucial developmental period, employing a sample with such a large age-range may mask differences that are present at a more specific age. Furthermore, there is recent evidence that the developmental trajectories of psychological resources such as self-esteem and mastery, which are likely to affect coping, are influenced by social class. Falci [56] found that high SES adolescents experience steeper gains in self-esteem and mastery compared to low SES adolescents during the high school years.

It is also possible that different variables will mediate the SES-stress relationship depending on the social, cultural and economic context. This may especially be the case with coping. If coping behaviour is in part socially determined, as the results of the present study suggest, then its measurement is likely to be sensitive to the social context in which it occurs. This presents researchers of adolescent stress and coping with a challenge. One approach would be to create context specific coping measures, which might make it easier to uncover relationships if they exist. The drawback of such an approach would be to muddy comparisons between studies and contexts.

While Wills et al. [36] did not include a measure of perceived stress in their study; they did test whether or not life events and coping mediated their observed relation between parental education and adolescents’ substance use. Life events were found to mediate this relation but coping variables did not.

Chen et al. [44] used two composite measures of SES, one combining parental education and occupation into a prestige-based SES measure, and one combining income and savings into an assets-based SES measure. Their cross-sectional study employed a sample of 15 to 19 year old adolescents, and tested the role of psychological interpretations of threatening and ambiguous stimuli in the relationship between low SES and physiological stress responses. They also collected data on negative and positive life events during the previous 6 months. Prestige SES was related to adolescents’ interpretations of ambiguous videos such that lower SES adolescents were more likely to interpret these as threatening than higher SES adolescents. This was not found when the analysis was repeated using the assets based SES measures. Lower prestige based SES was also related to higher heart rate reactivity and diastolic blood pressure while talking about the ambiguous video. Again, this was not found when using assets based SES. Threat interpretations partially mediated relationships between SES and physiological reactivity. General life events (e.g. a lack of positive events) partially explained the relationship between low SES and threat interpretations. Chen et al. [44] note that “…. the experience of repeated and unpredictable negative life events in low-SES children may lead them to develop patterns of thinking in which they come to expect the worst even in ambiguous circumstances.” Using this reasoning in the interpretation of our own results, this may in turn lead to a greater use of avoidance coping amongst low SES adolescents. This illustrates how complex the relations between life events (and other sources of stress), coping and perceived stress are likely to be in reality. One may question the meaningfulness of trying to discern whether differential exposure or differential impact best explains SES-differences in perceived stress, since coping behaviours are presumably formed through socialisation and learning processes throughout childhood, and these processes will in turn be influenced by exposure. In this way vicious cycles may develop, so that greater exposure to negative and uncontrollable life events leads to expectations of less control and more threat, which in turn promotes more avoidance, which reduces the likelihood of successfully dealing with problems, even when such problems are objectively controllable. In other words, greater exposure may lead with time to greater impact. It is important that ‘dispositional’ measures of coping strategies, such as those used here, should not be thought of purely as properties of the individual, since they partly reflect socialisation to differential social conditions that promote or restrict these strategies. As Baum et al. [11] note, many psychological factors “are products of environmental exposures associated with lower or higher SES, such as learned helplessness, a sense of powerfulness, or an orientation towards mastery and efficacy…”

### Using two measures of SES – advantages and disadvantages

Our results, in keeping with the studies discussed above, confirm the need to employ different indicators of SES in the same study. We found that both parental education and household income are related to adolescents’ perceived stress, while others have found this relation for parental education but not income [34]. On the other hand, we found that the amount of the SES – perceived stress effect that could be explained by life events and coping was to some extent dependent upon the measure of SES used. In general, mediation was strongest when parental education was used. An exception is seen in model 2 for girls (Table 4), where the choice of SES measure does not have much influence on the extent to which life events mediate the SES – perceived stress relation. The choice of SES measure influences results most clearly in model 3 for boys (Table 3). Here, coping is seen to mediate the relation between parental education and stress, but not the relation between household income and stress. The present study suggests that employing different indicators of SES could provide a means of examining gender differences in meditational processes connecting SES with stress.

We found that a clear social gradient in life events was apparent irrespective of SES-measure. Brady et al. [54] also found greater life event exposure in lower SES adolescents. Interestingly, different indices of SES were predictive of different types of life events. For example, greater assets (a composite measure of material resources) predicted fewer negative life events that were considered to be independent of respondent behaviour, and fewer total life events, while fathers’ education predicted fewer negative life events that were not independent of respondents’ behaviour. The study also included a measure of positive life events, but these were not related to any SES index.

### Strengths and limitations

Several limitations of our study need to be addressed. Firstly, given the cross-sectional nature of the study, the meditational analyses cannot conclude whether life events and coping operate in a causal fashion. Both could account for some of the variance in the SES-perceived stress relation. Given that major life events, such as parental divorce or death, were reported, it seems unlikely that present day stress levels would influence recall or actual event occurrence. With coping however, there is likely to be more reciprocity. As mentioned previously, a greater use of avoidance coping could conceivably be a consequence of higher stress levels as well as a cause. Our results do however indicate a need to further investigate the possibility of a mediating role for coping. A second potential limitation concerns the possibility of response bias. As might be expected, non-responders were more likely to have lower SES. However, since all SES-groups were well represented in the sample we consider any potential for response bias to be minimal. Ensuring sufficient numbers in each SES group was in part achieved via our strategy of gaining access to schools and asking pupils to fill out the questionnaires there, during class-time. Another possible limitation is that we only collected data on negative life events. Given Chen et al. [44] finding that lack of positive events, rather than a greater frequency of negative events, mediated the relation between SES and physiological stress reactivity, the present study could have been strengthened by measuring both types of events. A final possible limitation concerns the generalizability of our findings. As our sample we used a whole birth cohort from a well defined region in Denmark. The region is predominantly rural and relatively affluent. Denmark is one of the richest countries in the world. Because of a progressive tax system and a welfare state, that ensures free education and health services for everyone, material differences between rich and poor are relatively small. In less egalitarian societies one might expect SES differences in perceived stress to be larger. It was also mentioned above that different variables might mediate the SES-stress relationship depending on the social, cultural and economic context. Whether or not specific coping strategies, for example, are beneficial, is indeed likely to be very context dependent. The consequences of life events, such as parental divorce, in terms of changed life trajectories, chronic living conditions and future possibilities are also likely to be dependent upon the societal conditions in which they occur. Again, the Danish context of the present study seems more likely to weaken rather than intensify the ‘stressfulness’ of low SES, compared to countries with a less established welfare system.

The present study also has a number of strengths. Firstly we employ data from a large cohort study with a very high response rate. Secondly, since we use two independent sources of data, self-report questionnaire data combined with data from national registers, the relationships found between SES and perceived stress cannot be accounted for by common method variance. Thirdly, the use of national registers enabled a reliable and objective categorisation of participants into SES-groups. Fourth, because we use two different measures of SES, our study can contribute to the ongoing discourse regarding the nature of SES and its relation with stress. While income and education are related, our analyses suggest that they may exert an influence on stress via different mechanisms, and that these mechanisms to some extent vary according to gender.

## Conclusions

The main conclusion of this study is that negative life events during childhood and coping behaviour mediate the social gradient in perceived stress among 14–15 year old Danish adolescents. The higher level of perceived stress reported by lower SES adolescents is related to more exposure to negative life events and increased vulnerability to stress reflected by less adaptive coping. As far as we are aware, this is the first time that such a meditational role for coping has been found in this age-group, though there is very little research addressing this question. We have provided possible explanations for discrepant findings.

Adolescence is likely to be a formative period with regard to many psychological variables that potentially mediate the SES – stress relationship. There is a clear need for longitudinal research that can unravel the complex interplay between SES, gender, differential exposure, context dependent socialisation and learning processes, the development over time of coping and coping resources, and the long term effect of these relationships on stress reactivity. How are coping strategies developed during adolescence? In what ways are these strategies affected by SES and/or exposures to stressful conditions and events? Future research will benefit from employing different measures of SES in the same study. It will also be a good idea to include assessment of the influence of positive events and conditions on stress and coping, rather than focussing narrowly on negative events.

A greater understanding of how coping and coping resources are formed over time is important for preventative efforts. However, it would be too simplistic to conclude, on the basis of the present study, that the coping behaviours of low-SES adolescents need to be changed, e.g. that they should be encouraged to employ more active coping and less avoidance coping. If coping patterns are socially determined then it is the social determinants of coping that we need to uncover and alter, if lower SES groups are to have a better chance of dealing with the stressors they encounter.

## Competing interests

All authors declare that they have no competing interests.

## Authors’ contributions

DG conceived the idea for the paper. CH performed the statistical analyses. JHA provided supervision for the statistical analyses. DG, KR and CH jointly drafted the manuscript with the main part of the introduction and discussion written by DG and the main part of the methods and results section drafted by CH. Revisions were made by CH. All authors interpreted the data and approved the final manuscript.

## Pre-publication history

The pre-publication history for this paper can be accessed here:

http://www.biomedcentral.com/1471-2458/13/532/prepub
